# Stakeholder perspectives and requirements for environmental-data-driven capacity forecasting in stroke care: a national survey of German stroke units

**DOI:** 10.3389/fneur.2026.1918423

**Published:** 2026-09-01

**Authors:** Lino Braadt, Lucas Proell, Pablo F. F. Escrihuela Branz, Elke Hertig, Ludwig C. Hinske, Till Baldenius, Patrik Dröge, Christian Günster, Thomas Ruhnke, Yevgeniia Ignatenko, Christian Merkenschlager, Markus Naumann, Philip W. J. Raake, Katharina Sasse, Iñaki Soto-Rey, Bastian Wein, Hauke Schneider

**Affiliations:** 1 Department of Neurology and Clinical Neurophysiology, University Hospital Augsburg, Augsburg, Germany; 2 Department of Cardiology, Faculty of Medicine, University of Augsburg, Augsburg, Germany; 3 Regional Climate Change and Health, Faculty of Medicine, University of Augsburg, Augsburg, Germany; 4 Institute for Digital Medicine, University Hospital of Augsburg, Augsburg, Germany; 5 AOK Research Institute (WIdO), AOK-Bundesverband, Berlin, Germany; 6 Faculty of Medicine, Dresden University of Technology, Dresden, Germany

**Keywords:** atmospheric data, capacity forecast, exposome, monitoring system, resource allocation, stroke, stroke unit, survey

## Abstract

Stroke remains a major health burden in Europe, with rising absolute case numbers. Atmospheric factors such as air pollution and temperature extremes are increasingly recognized as stroke risk determinants. Many hospitals face growing staff shortages, highlighting the need for predictive management of stroke care resources. This study surveyed directors of German stroke units to explore current strategies, challenges, and the perceived usefulness of a digital, environmental data-driven forecasting tool for resource management. As part of the ALERT-ITS project, a cross-sectional, web-based survey was conducted among certified stroke units in Germany. The questionnaire was developed using the REDCap® software and pre-tested by senior neurologists. The survey was distributed with support from the German Stroke Society in June and July 2025. 58 valid responses were analyzed. Most respondents had over 15 years of clinical experience. High occupancy rates (>80%) were common, and nursing shortages were the leading cause of bed closures. Although 48.3% (*n* = 28) of stroke units were part of a stroke network, structured coordination of resources in such networks was rarely established: only about one in five networked units (21.4%, *n* = 6) exchanged capacity data at least weekly, and nearly half (46.4%, *n* = 13) never did so. Communication within networks for acute stroke care primarily relied on telephone contact (89.3%, *n* = 25), while digital tools (39.3%, *n* = 11) or video consultations (35.7%, *n* = 10) were less common. Key requirements for an atmospheric factor–based prediction tool included short forecast horizons of 24–48 h, a reliability threshold (>80%), and detection of increases of three to four beds. Overall, opinion on the need for a digital forecasting system was evenly divided [44.8% (*n* = 26) perceived a need, 44.8% (*n* = 26) did not, and 10.3% (*n* = 6) were undecided]. By integrating atmospheric factors with real-time clinical data to generate short-term demand forecasts, such systems could optimize resource allocation, enhance coordination among stroke units, and support timely, high-quality patient care. This even split underscores that clearly defined user requirements, rather than a broad consensus on demand, are the central contributions of this work.

## 1 Introduction

Stroke remains a major public health challenge in Europe. In 2019, stroke accounted for more than 8 million disability-adjusted life years in the European Union (EU-28) with age-standardized rates remaining broadly stable in recent years (1). However, the absolute numbers of incident strokes and stroke prevalence continue to rise, driven by population growth and increasing life expectancy (1), and European projections anticipate a further substantial increase in stroke cases and survivors over the coming decades (2).

In addition to established cardiovascular risk factors such as arterial hypertension, dyslipidemia, and diabetes mellitus, environmental factors are increasingly recognized as important determinants of stroke risk and prevention. This growing interest reflects a shift toward understanding the exposome, comprising the totality of environmental exposures throughout life and their impact on health, complementing the well-established role of the genome in individual health (3). Among these exposures, air pollution has emerged as particularly significant, having been described as the “world’s largest single environmental health risk” (4). Recent studies have demonstrated strong associations between stroke incidence and multiple atmospheric factors: air pollution and types of pollution (5), local temperature variations including rapid changes (6), extreme absolute temperatures during both daytime (7) and nighttime (8), and specific weather types (9). While effect sizes of single atmospheric factors are typically modest in isolation – for example an OR of 1.14 (95% CI 1.01–1.32) for stroke after extremely hot nights (8) or a 1.4% increase in stroke hazard per 1 °C during warm seasons (10) – the global heat-attributable burden has been intensifying over time (average annual percentage change +0.73%, 95% CI 0.37–1.10, between 1990 and 2019) (7). Importantly, individual factors capture only part of the picture, as combined exposures might compound. For example, temperature may interact with air pollution, potentially causing synergistic rather than only additive effects (11), or with humidity, potentially resulting in variable thermal load.

The rising number of stroke patients (1) coincides with a persistent shortage of medical staff in many hospitals, including both physicians and nurses (12). Without innovative approaches to resource allocation, the widening gap between the increasing number of stroke patients and limited healthcare capacities is likely to compromise the quality of stroke care. Proactive planning and implementation of predictive digital tools are therefore essential: by supporting measures such as adapted staffing during forecasted high-demand periods and anticipatory bed capacity planning, they could enhance stroke resource management while also optimizing healthcare efficiency and reducing costs.

The established links between atmospheric factors and stroke incidence offer opportunities not only for urban planning and political action (10) but also for practical clinical applications. While individual education about temperature extremes has proven effective regarding behavioral responses (13), the potential for systematic use of environmental data in healthcare settings remains largely untapped. Although weather forecasts are ubiquitous, their application to stroke risk prediction is still emerging. Recent work by Santhanam et al. demonstrates that machine learning can forecast stroke incidence using environmental data, even without air pollutants (14). Incorporating air quality data could further enhance predictive accuracy.

**Figure 1:**
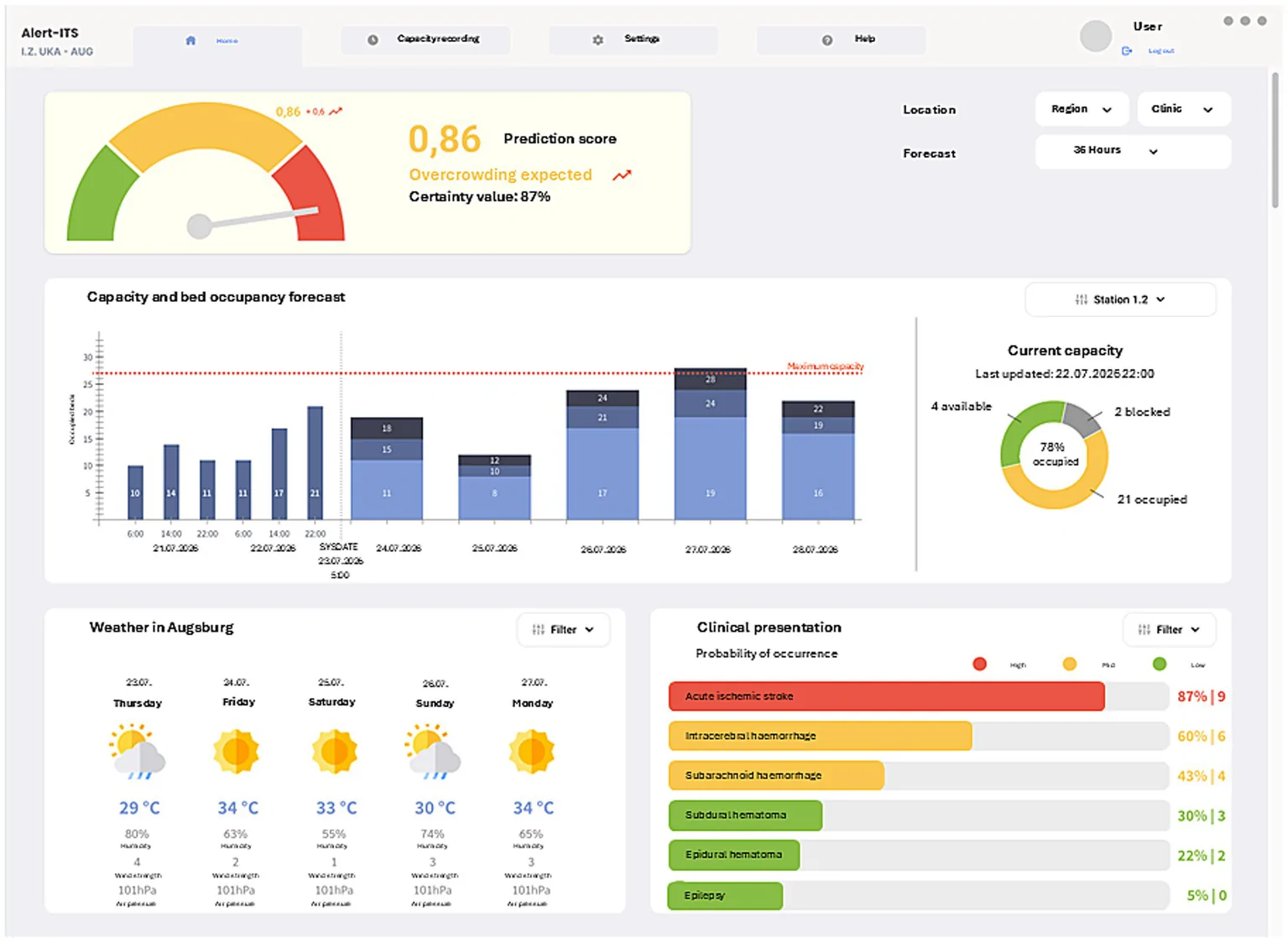
Illustrative user interface of the ALERT-ITS prototype, originally developed for intensive-care capacity forecasting (15) and shown here to convey the intended dashboard concept. All displayed values are synthetic and for illustration only. The multi-day capacity chart and weather panel reflect the current prototype build; for stroke-unit use, the tool is envisaged to operate at the 24–48 h horizon and bed-level resolution elicited in this survey.

Originally developed for intensive care unit capacity planning (15), the ALERT framework is being explored for possible extension to stroke care, motivated by the growing evidence on atmospheric-stroke associations. As a first step, and before any such tool is developed, we conducted a survey among certified German stroke units to understand stakeholder needs. The study aimed to (i) characterize current strategies for managing staffing and capacity shortages in stroke care, (ii) identify specific challenges in resource allocation during periods of peak demand, and (iii) assess stakeholders’ perceived need for, and requirements of, an atmospheric-factor-based capacity-forecasting tool. Here, we report the survey results, including the divided stakeholder opinion on the need for such a tool, and discuss their implications for whether and how such systems should be developed (Interface shown in Figure 1).

## 2 Materials and methods

### 2.1 Study setting and objectives

As part of the ALERT-ITS project (Development of a Prediction and Monitoring Model to Forecast Regionally Varying Intensive Care and Ventilation Demand Driven by Environmental Factors), we conducted a cross-sectional, web-based survey among certified stroke units across Germany. In Germany, stroke units are hospitals whose acute stroke care meets defined structural, staffing, and process standards audited under the certification program of the German Stroke Society (Deutsche Schlaganfall-Gesellschaft, DSG), which distinguishes regional and supra-regional stroke units. The study adopted an exploratory approach to examine current staffing and capacity planning practices in stroke care, as well as existing forms of collaboration and coordination within regional stroke networks. In addition, it explored stakeholders’ expectations regarding the use of digital tools to support clinical decision-making, capacity forecasting, and enhanced resource management.

The study was reviewed by the Ethics Committee of the Ludwig-Maximilians-Universität Munich, which, following review of the protocol including the associated stakeholder interviews, issued a favorable opinion, confirming no ethical or legal objections to the study (Project No. 24–0648; 30 September 2024). The study was prospectively registered with the German Clinical Trials Register (DRKS00035640). Reporting follows the CHERRIES (Checklist for Reporting Results of Internet E-Surveys) guidelines (16).

### 2.2 Questionnaire design and data collection

Building on previously published surveys of German stroke units and on interviews with neurologists at Augsburg University Hospital, the study team developed a questionnaire comprising several distinct thematic sections (17–19). These addressed the structural characteristics of stroke units, including organizational setup, current occupancy levels, and existing procedures for demand-oriented personnel planning. The questionnaire also examined the extent and quality of collaboration within regional stroke care networks. In addition, specific requirements and expectations regarding a predictive digital tool to support capacity forecasting and the efficient allocation of resources were addressed.

The questionnaire was developed using Research Electronic Data Capture (REDCap®) software (20, 21) and underwent comprehensive review by the project consortium. Prior to finalization, it was pre-tested with two local senior stroke neurologists. Feedback from this phase was incorporated to refine the instrument. The final survey comprised 83 questions, with an estimated completion time of approximately 20 min. The complete questionnaire is provided in the supplementary material (Supplementary file 1).

The survey was conducted in June and July 2025. The sampling frame comprised the 346 certified stroke units in Germany at the time of the survey; contact information for these units was compiled from publicly available sources, and all were directly invited by email. To broaden outreach, the invitation was additionally circulated through the email distribution list of the German Stroke Society (DSG), whose size and overlap with the direct invitations could not be determined. All identified individuals received an email invitation containing comprehensive information about the ALERT-ITS project, the purpose of the survey, and relevant data protection details. Participation was voluntary, and informed consent was obtained prior to accessing the questionnaire. To increase the response rate, two reminder emails were sent, the first two weeks after the initial invitation. No financial or material incentives were provided to respondents. Data collection and management were performed using the REDCap® platform, hosted at the University Hospital of Augsburg (20, 21).

### 2.3 Data processing and statistical evaluation

The survey closed four weeks after the second reminder. Anonymized data were exported from REDCap® and independently reviewed by two authors for plausibility. Two blank questionnaires were excluded from analysis. Partial responses were retained, with item non-response reported where applicable.

Categorical data were presented as frequencies and percentages of the total valid sample (*N* = 58). For conditional questions answered only by subgroups (e.g., network-specific questions), the relevant subgroup denominator is stated alongside the percentage at first mention, so that the reference population for each item is explicit. Ordinal data from Likert scales were summarized using median and interquartile range (Q1-Q3).

The normality of Likert scale responses was assessed using graphical inspection and Shapiro–Wilk test, which confirmed non-normal distributions. Therefore, the Kruskal-Wallis test was applied to examine differences in capacity management strategies across hospital types (Table 1). Statistical significance was set at *p* < 0.05, employing two-sided tests. Data were analyzed using R (https://www.r-project.org/) and RStudio 2025.05.0 + 496 (Posit Software, Boston, MA, US).

**Table 1:** Comparison of hospitals regarding feasibility of different measures to react to capacity shortages.

Characteristic	Measure	Total	Primary/secondary care hospital	Non-university maximum care hospital	Tertiary care hospital	University hospital	*p*-value
Adjustment of nurse-to-patient ratio	*n* (valid)	58	9	9	29	11	
	Mdn (Q1-Q3)	2.0 (2.0–3.8)	2.0 (2.0–4.0)	2.0 (2.0–3.0)	2.0 (2.0–3.0)	3.0 (2.0–3.5)	*p* = 0.731
Adjustment of physician-to-patient ratio	*n* (valid)	57	9	9	28	11	
	Mdn (Q1-Q3)	3.0 (2.0–4.0)	4.0 (2.0–5.0)	2.0 (2.0–4.0)	2.5 (2.0–4.0)	4.0 (2.5–4.0)	*p* = 0.284
Increase in total bed capacity	*n* (valid)	57	9	9	28	11	
	Mdn (Q1-Q3)	2.0 (1.0–2.0)	2.0 (1.0–2.0)	2.0 (2.0–2.0)	2.0 (1.0–2.2)	2.0 (2.0–4.0)	*p* = 0.137
Prospective bed blocking	*n* (valid)	58	9	9	29	11	
	Mdn (Q1-Q3)	2.0 (2.0–3.0)	2.0 (2.0–4.0)	2.0 (2.0–2.0)	2.0 (1.0–3.0)	2.0 (2.0–2.5)	*p* = 0.882
Cancellation of elective procedures	*n* (valid)	58	9	9	29	11	
	Mdn (Q1-Q3)	3.0 (2.0–4.0)	2.0 (2.0–4.0)	3.0 (2.0–4.0)	3.0 (2.0–4.0)	3.0 (2.0–4.0)	*p* = 0.807
Transfer of patients to partner facilities	*n* (valid)	58	9	9	29	11	
	Mdn (Q1-Q3)	4.0 (2.2–4.0)	4.0 (3.0–4.0)	3.0 (2.0–4.0)	3.0 (2.0–4.0)	4.0 (4.0–4.0)	*p* = 0.061
Coordination with internal referrers (e.g., emergency department)	*n* (valid)	58	9	9	29	11	
	Mdn (Q1-Q3)	4.0 (3.0–4.0)	4.0 (3.0–5.0)	4.0 (3.0–4.0)	4.0 (3.0–4.0)	4.0 (4.0–4.0)	*p* = 0.438
Coordination with external referrers (e.g., network hospitals)	*n* (valid)	58	9	9	29	11	
	Mdn (Q1-Q3)	4.0 (3.0–4.0)	4.0 (3.0–4.0)	4.0 (2.0–4.0)	4.0 (3.0–4.0)	4.0 (3.5–4.0)	*p* = 0.279
Reduction of length of stay (when feasible)	*n* (valid)	58	9	9	29	11	
	Mdn (Q1-Q3)	4.0 (4.0–4.0)	4.0 (4.0–4.0)	4.0 (4.0–4.0)	4.0 (4.0–4.0)	4.0 (3.5–4.5)	*p* = 0.949

Comparison of Primary/secondary care hospital vs. Non-university maximum care hospital vs. Tertiary care hospital vs. University hospital using Kruskal-Wallis test.

Response scale: 5-point Likert scale (1 = not feasible, 2 = rather not feasible, 3 = undecided, 4 = rather feasible, 5 = feasible).

Mdn, Median.

## 3 Results

### 3.1 Survey participants

A total of 60 stroke unit representatives submitted the questionnaire, of which two were excluded, leaving 58 valid responses. Relative to the 346 directly invited certified stroke units, this corresponds to a participation rate of 16.8% and a completion rate of 96.7% (58 of 60 submitted); unique first-page visits were not recorded, so a view rate could not be computed. Because the survey was additionally circulated through the DSG distribution list of unknown size, the true number invited is at least 346, and the 16.8% figure should therefore be interpreted as an upper-bound estimate of the response rate.

North Rhine-Westphalia provided the highest share of responses (22.4%, *n* = 13), followed by Bavaria (17.2%, *n* = 10) and Baden-Württemberg (15.5%, *n* = 9). Single responses (1.7%, *n* = 1 each) were received from Mecklenburg-Western Pomerania and Saxony-Anhalt, while Bremen, Saarland, and Schleswig-Holstein provided no answers. Two participants (3.4%, *n* = 2) did not disclose their federal state. Respondents were highly experienced overall: 70.7% (*n* = 41) reported more than 15 years of experience in stroke care, 27.6% (*n* = 16) reported 6–15 years, and only one (1.7%) reported 3–5 years of experience. In addition, all participants were either senior physicians (22.4%, *n* = 13) or served as heads/chief physicians (77.6%, *n* = 45) of their respective stroke units.

### 3.2 Stroke care resources and capacity-related limitations

Participating stroke units represented diverse hospital types: 50.0% (*n* = 29) from tertiary care hospitals, 19.0% (*n* = 11) from university hospitals, and 15.5% (*n* = 9) each from primary/secondary care hospitals and non-university maximum care hospitals. Bed capacity varied across stroke units, with the majority operating 10–12 beds (41.4%, *n* = 24), followed by 4–6 beds (24.1%, *n* = 14), 7–9 beds (22.4%, *n* = 13), and >12 beds (12.1%, *n* = 7). Most commonly, these stroke units were operated by 1–3 or 4–6 full-time physicians (39.7%, *n* = 23 each), less common were more full-time physicians, e.g., 7–9 (13.8%, *n* = 8), 10–12 (1.7%, *n* = 1) or even more than 12 physicians (5.2%, *n* = 3).

High occupancy rates characterized most stroke units: 36.2% (*n* = 21) reported 81–90% average occupancy, 34.5% (*n* = 20) exceeded 90%, while 25.9% (*n* = 15) operated at 71–80% capacity. Only one facility (1.7%) reported <70% occupancy, and one respondent (1.7%) did not specify. The majority of respondents (77.6%, *n* = 45) reported bed closure rates of 0–5%, while 22.4% (*n* = 13) indicated higher closure rates: 8.6% (*n* = 5) reported 6–10%, 6.9% (*n* = 4) reported 11–20%, two participants (3.4%) reported 21–30%, and one respondent (1.7%) each reported 31–40% or >40% of beds being closed. The leading cause of these closures (multiple answers possible, valid answers *n* = 21) was a shortage of nursing staff alone (90.5%, *n* = 19), followed by measures for patient isolation (14.3%, *n* = 3).

**Figure 2:**
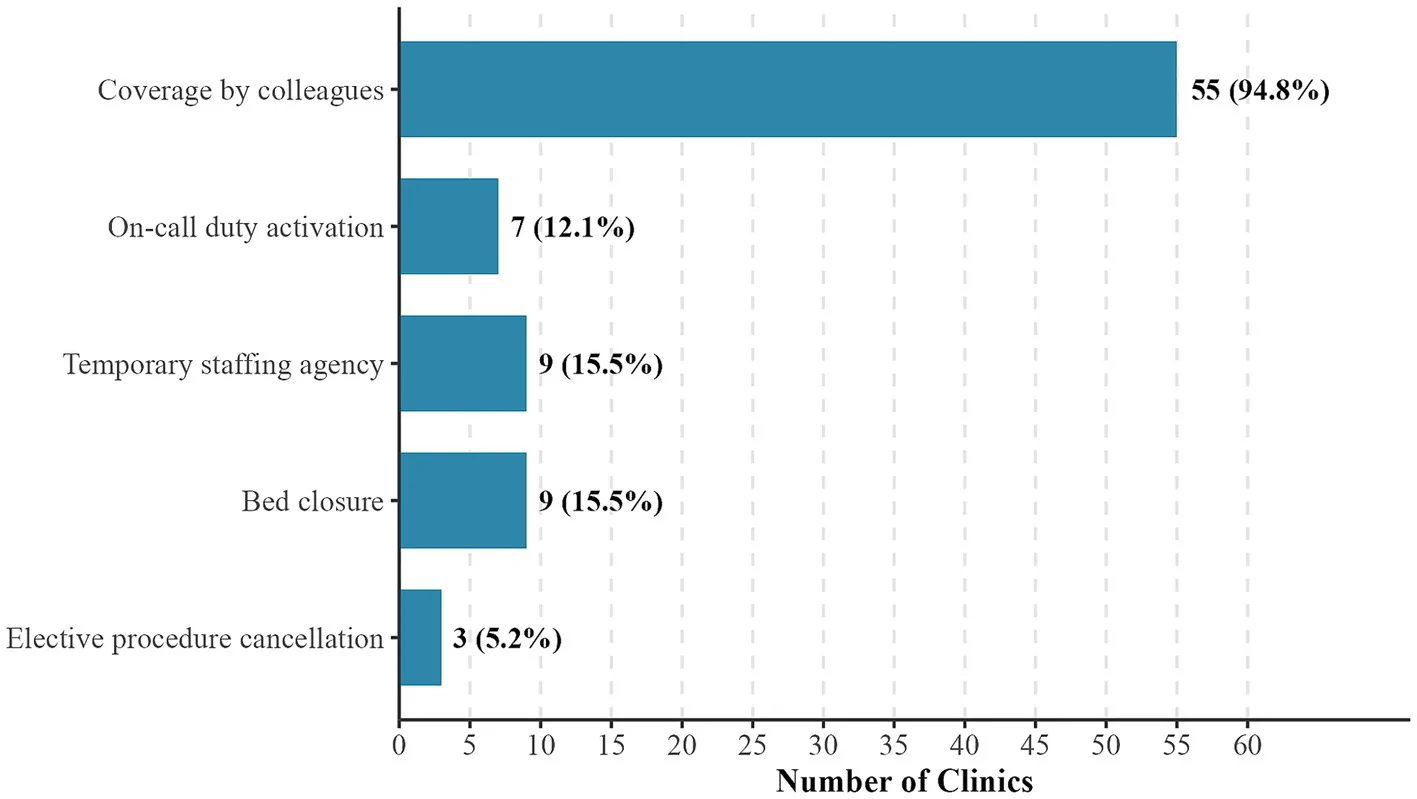
Response strategies of stroke units to acute physician staffing shortages. Multiple responses possible, *N* = 58.

To cope with these restricted capacities, various compensation mechanisms were applied. A total of 34.5% (*n* = 20) reported being able to use corridor beds, whereas 65.5% (*n* = 38) lacked this option. Among the 31 respondents who provided information on patient transfers, 87.1% (*n* = 27) reported the possibility of transferring patients to partnering clinics. Of the 22 stroke units that specified transfer frequency, 45.5% (*n* = 10) transferred fewer than 5% of patients to supra-regional stroke units, while 22.7% (*n* = 5) transferred 10–20%. Regarding discharge acceleration to rehabilitation clinics during capacity shortages, 27.6% (*n* = 16) confirmed this capability, 10.3% (*n* = 6) denied it, while 62.1% (*n* = 36) indicated limited availability of this option. Personnel shortages were primarily managed through colleague coverage across all hospital types (Figure 2). No significant differences emerged between hospital types regarding capacity management strategies (Table 1).

### 3.3 Role of stroke networks in coordination and capacity management

**Figure 3:**
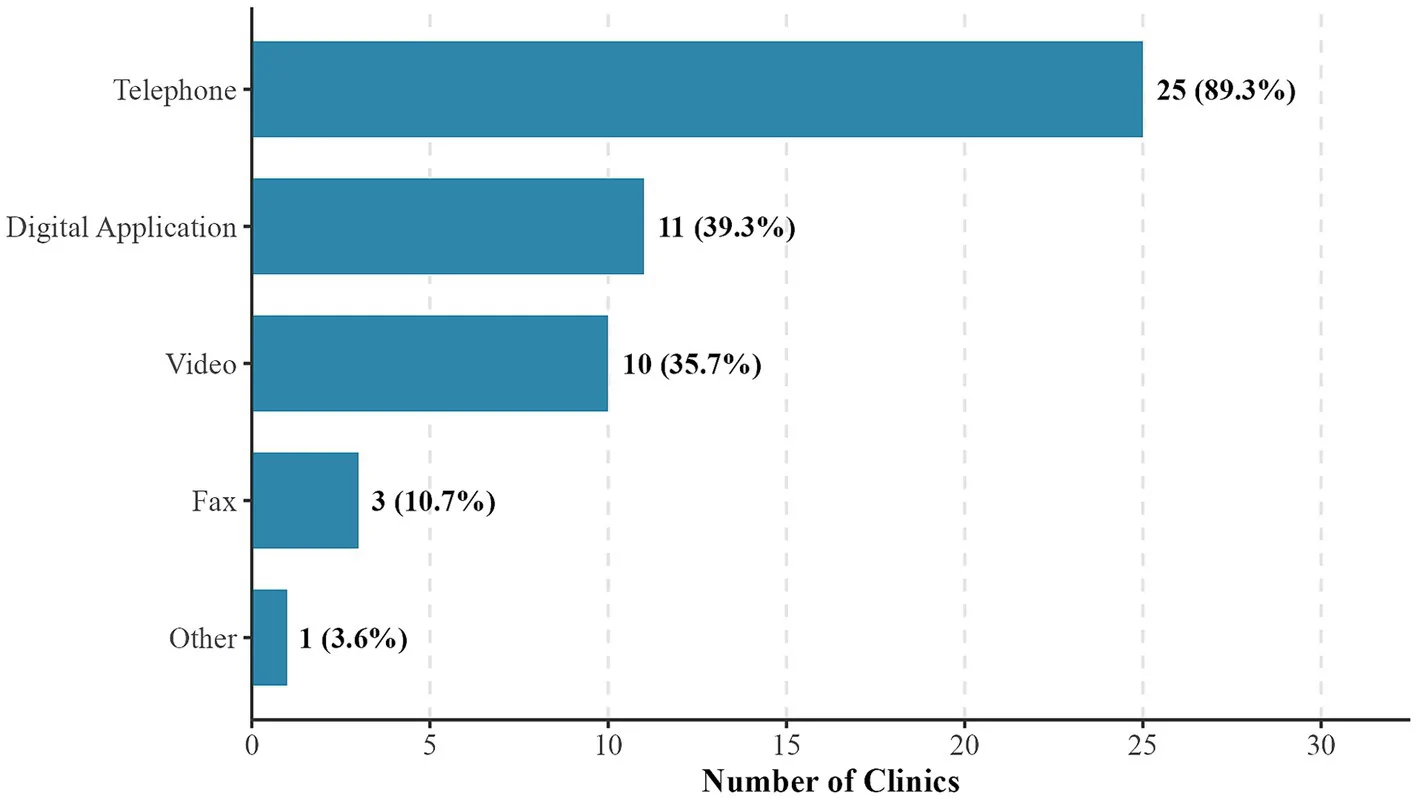
Communication methods for acute stroke care in stroke networks. Multiple responses possible (*n* = 28 stroke units participating in stroke networks).

Stroke networks provide efficient means of coordinating stroke care and managing patient flows. Among the surveyed stroke units, however, only 48.3% (*n* = 28) were part of a stroke network, while 50.0% (*n* = 29) were not, with one respondent abstaining. These networks were primarily used for telemedicine consultations in acute stroke care (92.9%, *n* = 26). Communication most often occurred via telephone (89.3%, *n* = 25), digital applications (39.3%, *n* = 11) and video consultations (35.7%, *n* = 10) (Figure 3).

Structured, regular information exchange regarding stroke care resources in networks was rarely established: 46.4% (*n* = 13) never shared such information, whereas only 10.7% (*n* = 3) exchanged data weekly and a further 10.7% (*n* = 3) daily, so that just 21.4% (*n* = 6) exchanged capacity data at least weekly. The majority reported the absence of a regional coordination center (82.1%, *n* = 23). However, patient transfers to supra-regional stroke units frequently occurred (*n* = 31), most commonly for thrombectomy (96.8%, *n* = 30), angiography (35.5%, *n* = 11), and complex neurological cases (67.7%, *n* = 21).

### 3.4 Stakeholder needs for stroke unit capacity forecasting and resource management

Digital solutions could hold considerable promise for supporting proactive and short-term capacity planning in stroke units and stroke networks. Even though only 15.5% (*n* = 9) of respondents currently rely on such applications to manage stroke unit bed occupancy and optimize resources, the vast majority (94.8%, *n* = 55) considered digital applications useful in the hospital setting.

In evaluation of an environmental data-based stroke prediction tool designed to forecast short-term stroke unit capacity needs, stakeholders expressed a strong preference for short prediction horizons. Forecasts of 24 h (27.6%, *n* = 16), 36 h (10.3%, *n* = 6), 48 h (32.8%, *n* = 19), or 72 h (22.4%, *n* = 13) were considered most useful, whereas longer periods beyond 72 h were rarely seen as relevant (5.2%, *n* = 3) and one respondent did not specify (1.7%). Regarding clinically meaningful lower thresholds for additional bed requirements, most respondents emphasized smaller increases: 62.1% (*n* = 36) considered 3–4 extra beds relevant, 19.0% (*n* = 11) regarded 1–2 beds as meaningful, and another 19.0% (*n* = 11) viewed demands of five or more additional beds as significant. This distribution was consistent across different stroke unit sizes (range: 53.8–71.4% for 3–4 beds). Finally, reliability emerged as a key prerequisite for practical implementation of digital forecast applications. Most participants (81.0%, *n* = 47) preferred forecasts to exceed an 80% probability of occurrence and many respondents therefore expressed the need to check the current prediction probability easily (48.3%, *n* = 28).

**Figure 4:**
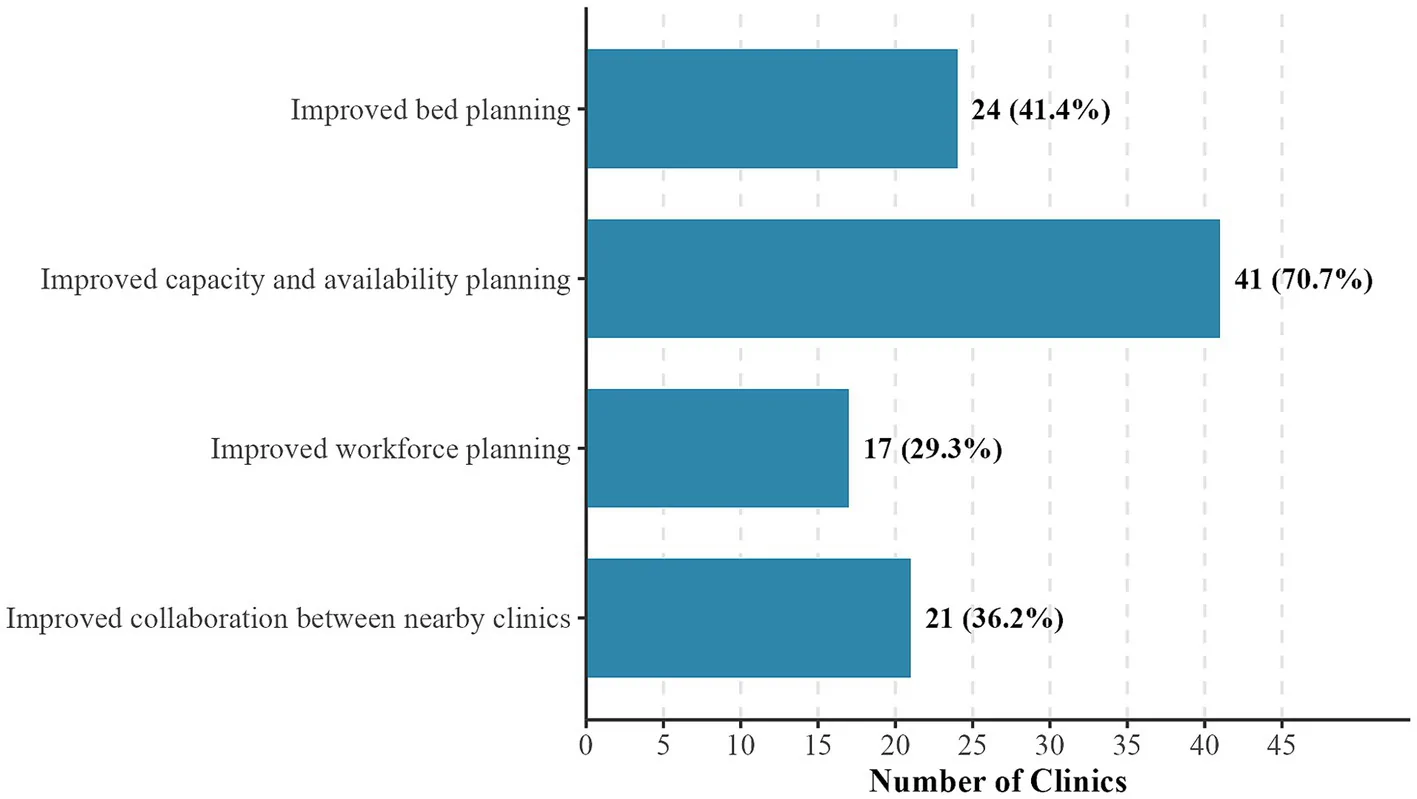
Expected advantages of a hospital capacity monitoring and prognosis system. Multiple responses possible; *N* = 58. Six respondents (10.3%) did not answer this question.

Beyond forecasting horizons and accuracy, stakeholders also emphasized the practical benefits such a tool could offer for day-to-day operations. A consolidated overview of available capacities was regarded as particularly valuable, with 70.7% (*n* = 41) indicating that it would substantially facilitate resource allocation. This, in turn, was seen as a means to enable more efficient deployment of limited specialist staff, pool nurses, or temporary agency workers (29.3%, *n* = 17). In addition, 36.2% (*n* = 21) highlighted that improved inter-hospital collaboration within a region could further optimize the distribution of stroke care resources (Figure 4).

In line with these anticipated benefits, usage frequency was expected to be high: 60.3% (*n* = 35) anticipated daily use and 24.1% (*n* = 14) even more frequent use; less frequent patterns were uncommon (multiple times per week: 6.9% (*n* = 4); weekly: 1.7% (*n* = 1); monthly: 3.4% (*n* = 2)). Two respondents (3.4%) did not specify a usage frequency.

Respondents highlighted several key functionalities as indispensable. Foremost, a general prediction score to estimate future bed demand was seen as essential (84.5%, *n* = 49). Also important was the simultaneous integration of capacity data, both from within the stroke unit (29.3%, *n* = 17) and especially from external stroke units (65.5%, *n* = 38), to enable a comprehensive overview across regional networks.

Finally, the system’s effectiveness was regarded as strongly dependent on the timeliness of data updates. While opinions varied, preferences most often pointed to updates every 8 h (29.3%, *n* = 17) or 12 h (25.9%, *n* = 15), with smaller groups favoring every 4 h (8.6%, *n* = 5), hourly (8.6%, *n* = 5) or daily updates (22.4%, *n* = 13). Three respondents (5.2%) indicated no specified preference. These findings highlight the need for a flexible yet standardized data infrastructure to support accurate and actionable forecasting.

Overall, the need for such a system was rated evenly among respondents: with six abstentions (10.3%), 44.8% (*n* = 26) indicated a perceived need, while 44.8% (*n* = 26) did not.

## 4 Discussion

To our knowledge, this is the first study to systematically assess stakeholder perspectives on integrating environmental data-based forecast models into hospital capacity planning. Notably, opinion on the need for such a system was evenly divided (44.8% perceived a need, 44.8% did not), even though the majority of stroke units already operate at very high occupancy rates.

These high baseline levels leave little buffer capacity to accommodate surge demand, reflecting structural strain on stroke unit resources, possibly even translating into care restrictions (22).

When facing additional capacity demands, stroke units employ several compensation strategies. At times of unplanned personnel shortage, for example due to sickness, colleagues routinely take over additional clinical responsibilities. Additionally, stroke units can transfer patients to partnering clinics within their stroke networks or, to some extent, accelerate discharge to rehabilitation facilities. While all these measures are regularly applied, some can rarely be implemented *ad hoc* and require at least some advance planning and communication.

This need for planning time aligns with respondents’ stated requirement for a prediction horizon of 24–48 h, which would improve the feasibility of implementing these measures. Respondents indicated that predictions of 3–4 additional beds would constitute a sufficiently large demand to trigger preparatory measures, a threshold that remained consistent across stroke unit sizes. Digital forecasting tools could support more proactive planning within this context, enabling stroke units to coordinate patient transfers, adjust staffing, and prepare for surge demand before capacity limits are reached.

Across all respondents, a majority anticipated daily use, suggesting that adopters would integrate the tool into routine planning. The minimum required prediction accuracy of >80% underscores the importance of reliable forecasts if they are to alter established routines. This threshold is appropriate given that false alarms could lead to unnecessary measures and erode trust in the system. The technical feasibility of meeting such reliability standards is supported by recent retrospective evidence: machine learning models successfully predicted stroke incidence using meteorological data (14). However, as no prospective operational forecasting tool has yet been implemented and validated in clinical practice, our stakeholder assessment provides critical insights into real-world requirements and constraints for translating such models into actionable capacity management systems.

This requirement must, however, be weighed against the predictive ceiling of atmospheric data. The environmental associations underlying such a tool are individually modest, for example an odds ratio of 1.14 for stroke after extremely hot nights and an approximately 1.4% increase in stroke hazard per 1 °C, and they represent population-level effects that explain only a small share of the day-to-day variation in stroke admissions. A further distinction is essential: forecasting a continuous rate or count, as demonstrated by Santhanam et al. for daily stroke incidence (14), is a materially easier task than issuing a categorical, bed-level alarm, that is, predicting whether a discrete increase of three to four beds will occur within 24–48 h at a probability exceeding 80%. At the level of an individual stroke unit, daily admissions are small counts subject to substantial stochastic variation, so an increment of three to four beds lies close to the intrinsic day-to-day noise; predictors with the modest effect sizes cited above are unlikely, in isolation, to resolve increments of this magnitude at the reliability stakeholders requested. Regional aggregation would improve the signal-to-noise ratio but reduce bed-level actionability for any single unit. In practice, much of the forecastable variation in admissions is carried by calendar, seasonal, and autoregressive structure, which suggests that atmospheric factors are more realistically an incremental input to a multimodal model, combined with real-time clinical and operational data, than a stand-alone predictor capable of meeting the stated threshold. The requirements elicited here should therefore be interpreted as a demanding benchmark against which future models must be prospectively validated, rather than as a capability that atmospheric-data prediction has already been shown to achieve. Establishing whether the desired >80% reliability at bed-level resolution is attainable, or whether it is partly aspirational, is a central objective for the subsequent development and validation phase of the ALERT framework.

Our findings should be positioned against existing capacity-management approaches. In routine German practice, real-time systems such as IVENA eHealth (Interdisziplinärer Versorgungsnachweis) display current hospital and stroke-unit capacities to emergency dispatchers and support immediate allocation of acute patients, but they provide a present-moment snapshot rather than a forward-looking forecast (23). Telestroke networks such as TEMPiS extend acute stroke expertise to hospitals without continuous on-site neurology and have been associated with higher thrombolysis rates and fewer transfers, yet they address access to clinical expertise and patient routing rather than anticipatory capacity planning (24). In parallel, statistical and machine-learning models have been developed to forecast hospital admissions and non-elective bed demand. A recent scoping review of 84 healthcare-capacity forecasting studies spanning five decades found ARIMA-type models to be the most common and reported a widespread lack of model validation and post-implementation monitoring across this field (25). Such models are typically generic rather than stroke-specific, operate at the level of a single institution rather than a regional network, and do not incorporate environmental drivers (26). The tool envisaged here differs in combining a prospective 24–48 h forecast, a stroke-specific and network-level perspective, and environmental data as an additional input; whether this combination can meet the requirements stakeholders defined remains, as discussed above, an empirical question.

Importantly, some respondents reported already using digital tools for bed capacity planning, suggesting that integration into existing workflows might be feasible and that prediction based on atmospheric factors could extend current planning capabilities. The emphasis on consolidated dashboards providing regional perspectives and inter-hospital coordination supports the concept of network-wide capacity management.

Capacity management strategies appeared broadly similar across hospital types, and we detected no significant differences between them. However, these subgroup comparisons rested on small strata (in some cases as few as nine units) and were therefore underpowered to exclude clinically meaningful differences; the absence of detectable differences is not evidence that none exist. This finding therefore offers only weak, provisional support for a single, common tool design, and the possibility of type-specific requirements cannot be excluded. Whether one design can adequately serve diverse users across German stroke units and networks should be regarded as a hypothesis for confirmation in a larger, adequately powered sample rather than as an established conclusion.

### 4.1 Strengths and limitations

The primary strengths of this study lie in the substantial professional experience of respondents (70.7% with >15 years’ experience) and representation across different hospital types.

The response rate of 16.8% (58 of 346 directly invited certified units) is lower than in other German neurological surveys with defined invitation denominators (e.g., 42.8% (*N* = 107) in Geisbüsch et al. (27)), likely reflecting questionnaire length; because the survey was additionally circulated via the DSG list of unknown size, this figure represents an upper-bound estimate of the true response rate. Nevertheless, respondents covered a broad spectrum of hospital types and stroke unit sizes. Regional representation, however, was uneven, with the highest participation from the most populous federal states and no response from three federal states. Some selection biases are likely: an overrepresentation of heads/chief physicians (77.6%, *n* = 45), absence of younger colleagues, and a self-selection of possibly digitally interested respondents (the survey title explicitly mentioned forecasting). This last point is important for interpreting the central finding: because the sample plausibly over-represents digitally interested respondents, the observed 44.8% endorsement is more likely to over-estimate than to under-estimate the need in the broader population of stroke units. An evenly divided opinion under these conditions should therefore be read as an upper-bound-leaning estimate of demand and argues against interpreting the result as strong stakeholder endorsement. Our survey targeted stroke-unit directors and senior physicians, who hold primary responsibility for capacity and staffing decisions; it did not, however, capture the perspectives of nursing staff or other allied health professionals. This is a relevant limitation, particularly because nursing shortages were identified as the leading cause of bed closures. Future studies should incorporate the views of nursing and multidisciplinary staff, whose input is central to resource planning in stroke care. In addition, the subgroup comparisons across hospital types were based on small and unequal strata (as few as nine units) and were statistically underpowered; the non-significant Kruskal–Wallis results should not be read as evidence of equivalence between hospital types.

Although a preliminary prototype has been established by the ALERT project team (Figure 1) (15), no functional stroke unit prototype was available, and cost–benefit considerations were not assessed. Therefore, future studies should pilot a functional prototype, validate adoption rates, and quantify economic implications.

### 4.2 Conclusion

Stakeholder opinion on the need for atmospheric factor-based stroke prediction tools was evenly divided, with 44.8% perceiving a need, 44.8% not, and 10.3% undecided; given a likely self-selection toward digitally interested respondents, this even split probably represents an upper-bound estimate of demand. The identified requirements, 24–48 h prediction horizons, >80% accuracy, sensitivity to 3–4 bed changes, and regional connectivity between stroke units, provide concrete benchmarks for such tools. Our ongoing work focuses on validating prediction algorithms and piloting implementation of digital applications to evaluate their impact on capacity management and patient care.

## Data Availability

The raw data supporting the conclusions of this article will be made available by the authors, without undue reservation.
