# Supplementary material for: Stakeholder perspectives and requirements for environmental-data-driven capacity forecasting in stroke care: a national survey of German stroke units
Source: Front Neurol. 2026 Sep 1;17:1918423. doi: 10.3389/fneur.2026.1918423 (PMC13586288; doi:10.3389/fneur.2026.1918423)
Supplement: Supplementary file 1 [file Supplementary_file_1.docx]

Supplementary Material

# 1 English version of the questionnaire

**ALERT-STROKE – Development of a prediction and monitoring model for regional forecasting of bed requirements based on environmental factors**

Dear Colleagues,

We are pleased that you are taking the time to participate in the stakeholder survey of the research project ALERT-STROKE.

Within the framework of the project, associations between weather patterns and environmental factors and the resulting increase in patient volume for specific disease entities are being analyzed. Based on the collected data, a prediction tool will be developed to forecast, in the short term and regionally, the additional demand for stroke unit beds attributable to environmental factors in an adult population. The aim is to develop a monitoring system that can quantitatively predict short-term additional resource needs, enabling regional providers to ensure sufficient healthcare capacity. Further information on the parallel ALERT-ITS project can be found at the following link. Information on data protection can be found in your participant information sheet (“Link to participant information”).

Capturing the requirements of interdisciplinary stakeholders represents a key component in the development of this monitoring system. For this purpose, we kindly ask you to provide information on the care structures of your stroke unit, resource allocation and planning processes, as well as your assessment of the requirements for a corresponding monitoring system.

All information provided in this survey is voluntary and anonymous. A withdrawal of participation after submitting the complete questionnaire is therefore not possible.

If you have any questions, please feel free to contact us at **lucas.proell@uk-augsburg.de** or by phone at **+49 821 400-2355**.

Completing the questionnaire will take approximately **15 minutes**.

For most questions, you can select one answer. In some cases, multiple answers are possible, or you will be asked to rate your response on a scale. For open-ended questions, you will be asked to provide one keyword reflecting your highest priority. The final part of the questionnaire is an evaluation of the monitoring system currently under development. Via the link provided, you will be directed to a prototype to which the questions refer.

Thank you very much in advance for your time and participation in our stakeholder survey!

I hereby confirm that I have understood all information provided. Under the study conditions described in the participant information sheet, I agree to participate in the survey described and to the processing of my information and data.

Clickable field: “YES”

| **Question No.** | **Question** | **Answer options** | **Type** |
| --- | --- | --- | --- |
| **Structural data of the Stroke Unit** | | | |
| **A1.1** | **How many years have you been working in stroke care?** | **0-3, 3-5, 5-10, 10-15, >15** | **Single choice** |
| **A1.2** | **In which federal state is your stroke unit located?** | **Baden-Württemberg, Bavaria, Berlin, Brandenburg, Bremen, Hamburg, Hesse, Mecklenburg-Western Pomerania, Lower Saxony, North Rhine-Westphalia, Rhineland-Palatinate, Saarland, Saxony, Saxony-Anhalt, Schleswig-Holstein, Thuringia** | **Single choice** |
| **A1.3** | **What position do you hold in your stroke unit?** | **Head of the stroke unit, Head physician (department chair), Senior physician/consultant, Specialist physician/attending physician, Resident physician** | **Multiple choice** |
| **A1.4** | **Which level of care is your hospital classified as?** | **Basic and standard care, Specialized and central care, Non-university maximum care, University maximum care** | **Single choice** |
| **A1.5** | **Does your hospital have departments of neuroradiology or neurosurgery?** | **Department of neuroradiology, Department of neurosurgery, Departments of neuroradiology and neurosurgery, None of the above** | **Single choice** |
| **A1.6** | **How many planned beds (= officially approved bed capacity defined in the hospital plan) does your hospital currently operate?** | **1-100, 101-250, 251-500, 501-1000, >1000** | **Single choice** |
| **A1.7** | **How many planned beds (= officially approved bed capacity defined in the hospital plan) does your stroke unit currently operate?** | **1-3, 4-6, 7-9, 10-12, >12** | **Single choice** |

| **Question No.** | **Question** | **Answer options** | **Type** |
| --- | --- | --- | --- |
| **Organizational and staffing structure – Physicians** | | | |
| **A2.1** | **Which specialty does the medical director/head physician of your stroke unit belong to?** | **Internal Medicine, Neurology** | **Single choice** |
| **A2.2** | **How many full-time physicians are employed in your stroke unit?** | **1-3, 4-6, 7-9, 10-12, >12** | **Single choice** |
| **A2.3** | **How do you respond to acute staff shortages (e.g., due to sick leave) among physicians?** | **Covering shifts by colleagues, calling in a pre-defined on-call service, hiring physicians via a temporary staffing agency, closing beds, Cancelling elective procedures in the coming days, Other (free text)** | **Multiple choice** |

| **Question No.** | **Question** | **Answer options** | **Type** |
| --- | --- | --- | --- |
| **Current occupancy situation on the Stroke Unit** | | | |
| **A3.1** | **What is the average occupancy rate of your stroke unit (proportion of occupied beds out of all non-closed beds)?** | **<70%, 71-80%, 81-90%, >90%** | **Single choice** |
| **A3.2** | **For approximately what proportion of patients is the 24-hour OPS Stroke code billed on your stroke unit?** | **<10%, 10-40%, 41-70%, >71%** | **Single choice** |
| **A3.3** | **For approximately what proportion of patients is the 24-hour OPS Stroke code billed outside the stroke unit (e.g., ICU)?** | **<10%, 10-40%, 41-70%, >71%** | **Single choice** |
| **A3.4** | **For approximately what proportion of patients is the 72-hour OPS Stroke code billed on your stroke unit?** | **<10%, 10-40%, 41-70%, >71%** | **Single choice** |
| **A3.5** | **For approximately what proportion of patients is the 72-hour OPS Stroke code billed outside the stroke unit (e.g., ICU)?** | **<10%, 10-40%, 41-70%, >71%** | **Single choice** |
| **A3.6** | **In acute situations, when the stroke unit is fully occupied, is overcapacity placement (e.g., hallway beds) possible on your stroke unit?** | **Yes, No** | **Single choice** |
| **A3.6.1 *(If A3.6 = “Yes”)*** | **How often do these overcapacity placements occur on average per month?** | **Never, On 1–2 days, On 3–5 days, On 6–10 days, On 11–15 days, On 16–20 days, On 21–31 days** | **Single choice** |
| **A3.7** | **How common are inappropriate admissions on your stroke unit (no stroke, e.g., epileptic seizure)?** | **Never, Rarely, Occasionally, Often, Always** | **Single choice** |
| **A3.8** | **On average, what percentage of the planned beds (= officially approved bed capacity defined in the hospital plan) of your stroke unit are not available for care / are closed?** | **0-5%, 5-10%, 11-20%, 21-30%, 31-40%, >40%** | **Single choice** |
| **A3.9** | **On how many days in the last month did you have to close beds on your stroke unit?** | **Never, On 1–2 days, On 3–5 days, On 6–10 days, On 11–15 days, On 16–20 days, On 21–31 days** | **Single choice** |
| **A3.9.1 *(If A3.9 ≠ “Never”)*** | **Why were these beds closed?** | **Nursing staff shortage only, Physician staff shortage only, Shortage of both physicians and nursing staff, Isolation requirements, Other reasons (free text)** | **Multiple choice** |
| **A3.9.1.1 *(If A3.9.1 = staff shortage)*** | **How do you cover the additional staffing needs that arise?** | **Float pool, On-call services, Locum/agency staff, Other (free text)** | **Multiple choice** |
| **A3.9.1.2 *(If A3.9.1 = staff shortage)*** | **How quickly do you cover the additional staffing needs that arise?** | **Within a few hours, With one day’s notice, With several days’ notice** | **Single choice** |

| **Question No.** | **Question** | **Answer options** | **Type** |  |
| --- | --- | --- | --- | --- |
| **Resource allocation within the stroke and hospital network** | | | | |
| **A4.1** | **In what capacity is your stroke unit involved in stroke care?** | **As a regional stroke unit (DSG certified), As a supraregional stroke unit (DSG certified), As a telemedicine-networked stroke unit (DSG certified), As a “comprehensive” stroke unit (DSG certified), As a non-certified stroke unit, In another capacity (free text), In none of the above capacities** | **Single choice** |  |
| **A4.1.1.1 *(If A4.1 = “supraregional stroke unit”)*** | **What proportion of patients are transferred from the stroke network (from regional stroke units), regardless of etiology (e.g., drip-and-ship)?** | **<5%, 5-10%, 10-20%, >30%** | **Single choice** |  |
| **A4.1.1.2 *(If A4.1 = “supraregional stroke unit”)*** | **Is it possible to transfer patients to another hospital within your network due to lack of capacity?** | **Yes, No** | **Single choice** |  |
| **A4.1.1.3 *(If A4.1 = “supraregional stroke unit”)*** | **What are the primary reasons for accepting patients transferred from an external facility?** | **Thrombectomy, Angiography, Complex neurological case, Other (free text)** | **Multiple choice** |  |
| **A4.1.1.4 *(If A4.1 = “supraregional stroke unit”)*** | **What are the primary reasons for accepting patients transferred from an external facility?** | **Thrombectomy, Angiography, Complex neurological case, Other (free text)** | **Multiple choice** |  |
| **A4.1.2.1 *(If A4.1 = “regional stroke unit”)*** | **What proportion of patients do you transfer to a supraregional stroke unit, regardless of etiology?** | **<5%, 5-10%, 10-20%, >30%** | **Single choice** |  |
| **A4.1.2.2 *(If A4.1 = “regional stroke unit”)*** | **Is there an option to transfer patients elsewhere within your network if transfer to a supraregional stroke unit is not possible due to limited capacity?** | **Yes, No** | **Single choice** |  |
| **A4.1.2.3 *(If A4.1 = “regional stroke unit”)*** | **Does a specialist in neurology work on your stroke unit?** | **No, Only on weekdays/during daytime hours, Available on site as a consulting physician, Permanently** | **Single choice** |  |
| **A4.2** | **Is your hospital part of a telemedicine stroke network?** | **Yes, No** | **Single choice** |  |
| **A4.2.1 *(If A4.2 = “Yes”)*** | **How often are you involved in telemedicine consultations in patient care?** | **Several times daily, Daily, Every 2 days, Every 3 days, Every 4–6 days, Weekly, Less than once per week, Never** | **Single choice** |  |
| **A4.2.2 *(If A4.2 = “Yes”)*** | **How are communication and coordination of patient transfers between your hospital and other centers within your stroke network handled at first contact?** | **Contact via a central digital platform/application, Telephone contact, Video contact, Fax contact, Other (free text), No specific communication** | **Multiple choice** |  |
| **A4.2.3 *(If A4.2 = “Yes”)*** | **How often is there an exchange of bed capacity information between the stroke units within the stroke network?** | **Daily, Every 2 days, Every 3 days, Every 4–6 days, Weekly, Less than once per week, Never** | **Single choice** |  |
| **A4.2.3 *(If A4.2 = “Yes”)*** | **Through which channels does this exchange take place?** | **Contact via a central digital platform/application, Telephone contact, Video contact, Fax contact, Other (free text), No specific communication** | **Multiple choice** |  |
| **A4.2.4 *(If A4.2 = “Yes”)*** | **Is there a supraregional coordination/dispatch center within your stroke network?** | **Yes, No** | **Single choice** |  |
| **A4.2.3.1 *(If A4.2.4 = “Yes”)*** | **Does this coordination/dispatch center have access to your current bed capacities?** | **Yes, No** | **Single choice** |  |
| **A4.2.3.2 *(If A4.2.4 = “Yes”)*** | **Does this coordination/dispatch center have authority to direct and manage the stroke unit capacities of the other hospitals?** | **Yes, No** | **Single choice** |  |
| **A4.3** | **Do you use digital/telemedicine tools in your clinical practice that are intended to facilitate coordination and bed planning within your stroke unit or within the stroke network?** | **Yes (free text), No** | **Single choice** |  |
| **A4.4** | **What challenges and barriers do you see in implementing telemedicine networking?** | **Technical issues, Data protection and IT security, Lack of trained staff, Funding and costs, Staff acceptance, Other (free text)** | **Multiple choice** |  |
| **A4.5** | **In addition to the stroke unit, does your hospital have an intensive care unit (ICU) and/or intermediate care unit (IMC)?** | **Yes, No** | **Single choice** |  |
| **A4.5.1 *(If A4.5 = “Yes”)*** | **Is this unit / are any of these units neurologist-led?** | **Yes, No** | **Single choice** |  |
| **A4.7** | **What would you change or improve about collaboration within your stroke network?** | **Free text field** | **Single choice** | |
| **A4.8** | **How often do you transfer patients directly from your stroke unit to a rehabilitation facility?** | **Never, Rarely, Occasionally, Often, Always** | **Single choice** | |
| **A4.9** | **Are there ways to speed up transfers to a rehabilitation facility?** | **Yes, Hardly, No** | **Single choice** | |

| **Question No.** | | **Question** | | **Answer options** | **Type** |
| --- | --- | --- | --- | --- | --- |
| **Capacity adjustments** | | | | | |
| In numerous publications, associations have been described between environmental factors/extreme weather events and diseases that require treatment on a stroke unit or in an intensive care unit. As mentioned in the introductory section, we are currently developing a prediction score that is calculated on a daily basis and forecasts the additional stroke unit bed demand attributable to environmental factors for a defined time period. | | | | | |
| **A5.1** | | **At what confidence level would you trust the prediction score and adjust your stroke unit capacity planning accordingly?** | | **50-70%, 71-80%, 81-90%, >90%** | **Single choice** |
| **A5.2** | | **Which forecast horizon would be relevant for your clinical practice?** | | **24 hours, 36 hours, 48 hours, 72 hours, 5 days, 7 days, >7 days** | **Single choice** |
| **A5.3** | | **Starting at what expected additional bed demand over the selected forecast period (A5.2) would such a score be relevant for your clinical practice?** | | **1–2 beds, 3–4 beds, 5–6 beds, 7–8 beds, 8–10 beds, 10–15 beds, >15 beds** | **Single choice** |
| In the following questions, please assume that the developed score meets the confidence level you selected in the previous questions, complies with the chosen forecast horizon, and exceeds the selected bed threshold. | | | | | |
| **A5.4** | | **How much lead time would you need to provide additional bed capacity?** | | **12 hours, 24 hours, 48 hours, 3–4 days, 5–6 days, >7 days** | **Single choice** |
| **A5.5** | | **Which factors would most likely limit an increase in capacity in your stroke unit?** | | **Shortage of physicians, Shortage of nursing staff, Shortage of both physicians and nursing staff, Spatial constraints (overall bed capacity), Other factors (free text)** | **Multiple choice** |
| **A5.6** | | **How would you provide additional bed capacity in your hospital for the coming days?** | | **Proactive bed closures (reservation of beds), Cancelling elective procedures, Increasing capacity by adding staff, Adjusting staffing ratios (physician-to-patient), Adjusting staffing ratios (nurse-to-patient), No capacity increase possible, Internal transfers, External transfers, Shortening length of stay (if possible), Other measures (free text)** | **Multiple choice** |
| **A5.7** | | **Who do you consider to be the key decision-makers for necessary capacity adjustments in response to a forecasted increase in patient volume over the coming days?** | | **Hospital management, Head physician/department chair, Medical director of the stroke unit, Nursing director/lead nurse of the stroke unit, Senior physicians/consultants, Resident physicians, Other decision-makers (free text)** | **Multiple choice** |
| **A5.8** | | **What additional information would be of interest to you to create additional bed capacity?** | | **Free text field** | **Free text field** |
| The following strategies are intended to help mitigate additional capacity demand. Please rate each strategy according to its feasibility for implementation in your stroke unit. | | | | | |
| **A5.9.1** | **Adjustment of nurse-to-patient ratio** | | **Not possible, Rather not possible, Undecided, Rather possible, Easily possible** | | **Likert scale** |
| **A5.9.2** | **Adaptation of physician-to-patient ratio** | | **Not possible, Rather not possible, Undecided, Rather possible, Easily possible** | | **Likert scale** |
| **A5.9.3** | **Increase in total bed capacity** | | **Not possible, Rather not possible, Undecided, Rather possible, Easily possible** | | **Likert scale** |
| **A5.9.4** | **Prospective bed blocking** | | **Not possible, Rather not possible, Undecided, Rather possible, Easily possible** | | **Likert scale** |
| **A5.9.5** | **Cancellation of elective procedures** | | **Not possible, Rather not possible, Undecided, Rather possible, Easily possible** | | **Likert scale** |
| **A5.9.6** | **Transfer of patients to partner facilities** | | **Not possible, Rather not possible, Undecided, Rather possible, Easily possible** | | **Likert scale** |
| **A5.9.7** | **Coordination with internal referrers (e.g., emergency department)** | | **Not possible, Rather not possible, Undecided, Rather possible, Easily possible** | | **Likert scale** |
| **A5.9.8** | **Coordination with external referrers (e.g., network hospitals)** | | **Not possible, Rather not possible, Undecided, Rather possible, Easily possible** | | **Likert scale** |
| **A5.9.9.** | **Reduction of length of stay (when feasible)** | | **Not possible, Rather not possible, Undecided, Rather possible, Easily possible** | | **Likert scale** |

| **Question No.** | **Question** | **Answer options** | **Type** |
| --- | --- | --- | --- |
| **Requirements for a monitoring system** | | | |
| The prediction score is intended to be integrated into a monitoring system in the form of a web-based app. The application and additional potential features will be evaluated below. | | | |
| **A6.1** | **On which device would you prefer to use the system?** | **PC, Smartphone, Tablet** | **Multiple choice** |
| **A6.2** | **Which of the following functions would be of highest priority for you and should therefore be visible on the start page? *(select 4 items)*** | **Current summarized prediction score including the resulting additional bed demand, Current confidence level of the prediction score, Capacity data of all stroke units within the stroke network, Capacity data of your own stroke unit, Current weather data, Weather forecast data and outlook on potential extreme weather events, Warning level model combining the prediction score with current capacity and weather data** | **Select 4 items** |
| **A6.3** | **Are there any other functions that would be essential for you but have not been mentioned so far?** | **If “Yes”: free text field; No** | **Single choice + free text** |
| **A6.4** | **As a user, to which data would you restrict access?** | **Hospital-related data (e.g., capacity input, capacity data, clinical coordination contacts), Weather- and environment-related data, Disease-related data (prediction score, expected disease entities), No restrictions** | **Multiple choice** |
| **A6.5** | **How would you prefer to be informed about warning levels and potential changes in expected bed demand?** | **SMS, Email, Push notification in the app, No preference** | **Single choice** |
| **A6.6** | **The application will include capacity data for stroke units and intensive care units. How often would you consider data entry to be necessary at a minimum?** | **Hourly, Every 4 hours, Every 8 hours, Every 12 hours, Every 24 hours, No preference** | **Single choice** |
| **A6.6.1** | **Would your choice in A6.6 depend on the predictive accuracy/confidence level (see question A5.1)?** | **Yes, No** | **Single choice** |
| **A6.8** | **How often would you use such an app?** | **Several times daily, Daily, Several times per week, Weekly, Several times per month, Monthly** | **Single choice** |
| **A6.9** | **Do you find digital systems helpful in everyday hospital practice?** | **Yes, No** | **Single choice** |

| **Question No.** | **Question** | **Answer options** | | **Type** |
| --- | --- | --- | --- | --- |
| **Evaluation of the click dummy (prototype** | | | | |
| **System description:** Please open a prototype of our system and test it: **LINK**.  After you have explored the prototype (the so-called *click dummy*), we kindly ask you to answer the following questions: | | | | |
| **A8.1** | **The presented data are displayed in an intuitive and understandable way.** | | **Strongly disagree (1), Disagree (2), Neutral (3), Agree (4), Strongly agree (5)** | **Single choice** |
| **A8.2** | **The use and learnability of the technology is easy.** | | **Strongly disagree (1), Disagree (2), Neutral (3), Agree (4), Strongly agree (5)** | **Single choice** |
| **A8.3** | **Based on the description above, the system works as I expected it to.** | | **Strongly disagree (1), Disagree (2), Neutral (3), Agree (4), Strongly agree (5)** | **Single choice** |
| **A8.4** | **I find the operation very cumbersome.** | | **Strongly disagree (1), Disagree (2), Neutral (3), Agree (4), Strongly agree (5)** | **Single choice** |
| **A8.5** | **I can very well imagine using the system regularly.** | | **Strongly disagree (1), Disagree (2), Neutral (3), Agree (4), Strongly agree (5)** | **Single choice** |
| **A8.6** | **Do you see a need for such a system in your stroke unit?** | | **Yes, No** | **Single choice** |
| **A8.7** | **What advantages do you see in using the planned monitoring system?** | | **Improved bed planning, Improved capacity and availability planning, Improved workforce planning, Improved collaboration between nearby clinics** | **Multiple choice** |
| **The following items address characteristics of the system. They consist of pairs of opposite terms. The steps between the opposites are represented by circles. By selecting one of these circles, you can indicate which term you agree with more.**  **Example: With this rating, you indicate that you consider the product to be *more attractive than unattractive*:**  **Attractive** **Unattractive** | | | | |
| **Obstructive Supportive** | | | | **Single choice** |
| **Complicated Easy** | | | | **Single choice** |
| **Inefficient Efficient** | | | | **Single choice** |
| **Confusing Clear/ Well-structured** | | | | **Single choice** |
